# Effect of recombinant PDGF-BB on bone formation in the presence of β-tricalcium phosphate and bovine bone mineral matrix: a pilot study in rat calvarial defects

**DOI:** 10.1186/s12903-016-0210-3

**Published:** 2016-05-04

**Authors:** Eloá R. Luvizuto, Stefan Tangl, Toni Dobsak, Karoline Reich, Reinhard Gruber, Celso K. Sonoda, Roberta Okamoto

**Affiliations:** Department of Surgery and Integrated Clinic, Araçatuba Dental School, UNESP-Univ Estadual Paulista, São Paulo, Brazil; Karl Donath Laboratory for Hard Tissue and Biomaterial Research, Division of Oral Surgery, Medical University of Vienna, Vienna, Austria; Austrian Cluster for Tissue Regeneration, Vienna, Austria; Department of Preventive, Restorative and Pediatric Dentistry, School of Dental Medicine, University of Bern, Bern, Switzerland; Department of Oral Biology, Medical University of Vienna, Vienna, Austria; Department of Anatomy, Araçatuba Dental School, UNESP-Univ Estadual Paulista, São Paulo, Brazil

**Keywords:** Bone substitutes, Bone formation, Bone regeneration, Bone augmentation, Platelet-derived growth factor-BB, Rat calvaria defects, β-tricalcium phosphate, Demineralized bovine bone mineral, Histomorphometry, Graft consolidation

## Abstract

**Background:**

Supplementation of bone substitutes with recombinant platelet-derived growth factor-BB (PDGF-BB) can enhance bone regeneration. The aim of the study was to evaluate the effect of PDGF-BB on bone formation in the presence of β-tricalcium phosphate and bovine bone mineral matrix in a rat calvaria defect model.

**Methods:**

The authors examined 5 mm rat calvarial defects treated with β-tricalcium phosphate (TCP) or demineralized bovine bone mineral (DBBM) with and without 0.3 mg/ml recombinant PDGF-BB. Calvaria defects were randomly divided into the following treatment groups (*n* = 5); TCP; TCP plus PDGF-BB; DBBM; DBBM plus PDGF-BB; and untreated empty control. After 45 days, bone formation was evaluated by histomorphometry and fluorescence microscopy.

**Results:**

The authors report that the area of newly formed bone was similar between the empty controls and the two bone substitutes, TCP and DBBM. Supplementation of TCP and DBBM with PDGF-BB had no significant impact on bone formation. Fluorochrome staining revealed no visible changes in the pattern of bone formation in defects filled with TCP and DBBM, irrespective of PDGF-BB. Furthermore, supplementation with PDGF-BB did not influence biomaterial degradation.

**Conclusions:**

The authors concluded that PDGF-BB had no impact on bone formation and degradation of bone substitutes in the respective rodent models. Thus, possible beneficial effects of PDGF-BB may require other model situations.

## Background

Biomaterials can be supplemented with growth factors to enhance the natural process of bone formation [1]. Bone formation is part of a sequential process initiated by a blood clot that is formed and organized into a granulation tissue before woven bone spans the defect space [2]. Woven bone provides a scaffold for parallel-fibred bone deposition, and finally bone remodeling dominates the scene, resulting in lamellar bone. Recombinant platelet-derived growth factor-BB (PDGF-BB) can support the process of bone formation, presumably by serving as a mitogen and chemoattractant for mesenchymal cells and by stimulating inflammatory cells such as macrophages to secrete growth factors [3–5]. This concept has led to a series of preclinical and clinical studies on the beneficial effects of PDGF-BB when combined with bone substitutes that provide a surface for the newly formed bone to grow from the edges into the center of the defect.

Preclinical studies have suggested that PDGF-BB in combination with deproteinized bovine and equine bone blocks can regenerate significant amounts of bone in severe mandibular ridge defects [6, 7]. Similar approaches have shown that PDGF-BB can accelerate fracture healing in geriatric osteoporotic [8] and diabetic rats [9], and enhance healing after bone distraction [10]. Thus, there is accumulating evidence from preclinical research that PDGF-BB can support bone formation. From a clinical perspective, the FDA has approved the combination of PDGF-BB and β-tricalcium phosphate (TCP) for the treatment of periodontal defects (GEM21S®) [11]. Moreover, locally administered PDGF-BB and TCP (Augment®) have been investigated in acute wrist fractures [12] and proved to support hindfoot and ankle fusions [13]. Thus, supplementation of TCP with PDGF-BB is a favorable strategy in regenerative dentistry, orthopedics and traumatology.

Critical-size rat calvarial defect models have been used to test PDGF-BB together with a chitosan carrier [14, 15]. However, whether TCP and demineralized bovine bone mineral (DBBM) supplementation with PDGF-BB is capable of overcoming the weak osteogenic potential of the rat calvarium remains to be tested. The aim of this pilot study was to evaluate the effect of PDGF-BB on bone formation in rat calvarial defects augmented with TCP or DBBM after 45 days, by means of histomorphometry and fluorescence microscopy.

## Methods

### Study design and ethics

Twenty-five Wistar rats (90 days old), acquired from the Animal Center of São Paulo State University were maintained at a temperature of 22 °C in a 12-h light/ 12-h dark cycle with free access to water and rodent food. A total of 25 calvarial defects (5-mm-diameter, one defect per animal) were randomly divided into 5 treatment groups, with a total of 5 defects per treatment group (*n* = 5). The treatment groups were as follows: [1] 500–1000 μm β-tricalcium phosphate (TCP) (Cerasorb®M, Curassan Ltd, Germany); [2] TCP plus 0.3 mg/ml of rPDGF-BB (R&D Systems, Inc., Minneapolis, MN, USA); [3] 250–1000 μm demineralized bovine bone mineral matrix (DBBM) (0.25 mm-1 mm Bio-Oss®, Geistlich Pharma Group Ltd, Wolhusen, Switzerland); [4] DBBM plus PDGF-BB; and [5] an untreated empty control (EC). The present study complied with the principles of laboratory animal care and national laws on animal use, and the study was authorized by the Animal Research Ethics Committee of the Araçatuba Dental School, UNESP-Univ Estadual Paulista, São Paulo, Brazil (protocol # 02402–2012).

### Surgical procedures and fluorochrome labeling

After general anesthesia with xylazine (0.03 ml/100 g body weight [bw]/intraperitoneal [ip]; Dopaser® Laboratories Calier SA, Barcelona, Spain) and ketamine (7 ml/kg bw/ip; Fort Dodge Saúde Animal Ltd, Brazil), the animal skulls were shaved and disinfected with polyvinylpyrrolidone iodide (Indústria Química e Farmacêutica Rioquímica Ltd, Brazil). By using an aseptic technique, an incision was made through the skin of the calvaria and periosteum, and full-thickness flaps were reflected. Under copious sterile saline irrigation, one 5-mm-diameter bone defect was prepared with a trephine bur (3i Implant Innovations, Inc., Palm Beach Gardens, USA) in each animal. The defect was treated as described above before the periosteum was repositioned and sutured with polylactic acid sutures (Vycril 5.0, Ethicon, Johnson Prod., São José dos Campos, Brazil). The skin was closed with nylon sutures (Nylon 5.0, Mononylon, Ethicon). All animals received a single dose of 20.000 UI of penicillin G benzathine (Pentabiótico, Veterinário Pequeno Porte, Fort Dodge Saúde Animal Ltd, Campinas, Brazil) by intramuscular injection. Rats received 20 mg/kg body weight of calcein and alizarin – soluble in saline solution - (Sigma Chemical, St. Louis, MO, USA) by intramuscular injection on the 15th and 30 th postoperative day, respectively. The rats were euthanized by anesthetic overdose after the 45th postoperative day.

### Sample processing and histomorphometric analysis

The relevant part of the skull was removed and fixed in neutral buffered 4 % formalin solution. The samples were dehydrated in ascending grades of alcohol and embedded in light-curing resin (Technovit 7200 VLC + BPO, Kulzer & Co., Hanau, Germany). The blocks were processed with the Exakt Cutting and Grinding Equipment (Exakt Apparatebau, Norderstedt, Germany) in accordance with a standardized method [16]. Sections were prepared parallel to the sagittal suture through the center of the defect and reduced to a thickness of approximately 40 μm before being stained with Levai-Laczko dye. One sample per animal, the centermost section, was subjected to blinded histomorphometric analyses. Stained sections were examined by light microscopy, and digital images were captured with the Olympus DotSlide system 2.4 (Olympus, Tokyo, Japan). Single pictures were assembled and stitched to one another to generate large overview images with a resolution of 3117 pxl/mm. For the final histomorphometric evaluation this initial resolution was reduced to 1559 pxl/mm. A set of rules for the classification of bone and bone substitute materials was created with the Definiens Developer XD 2.1 software (Definiens, Munich, Germany) based on color and morphological differences and the results were manually controlled and corrected in the Photoshop program (Adobe Photoshop, Adobe, San Jose, CA, USA). Analysis was restricted to the gap region (TAr) between the two edges of the drilled hole. With the histomorphometry software (Definiens Developer XD 2.1, Munich, Germany) the areas of newly formed bone (nBAr) and bone substitute material (BSAr) were measured in mm^2^. From these primary measurements the percentages of newly formed bone (nBAr/TAr) and bone substitute material (BSAr/TAr) within the total region of interest (TAr) were calculated. Qualitative fluorochrome analyses were performed with use of the epifluorescence mode of a Nikon Microphot-FXA microscope (Nikon, Tokyo, Japan).

### Statistics

Data were analyzed by using permutation tests based on t-tests; *p*-values were adjusted for multiple testing by means of the step-down minP procedure. Significant difference was assigned at the level of *p* < 0.05. The R version 3.1.0 (R Core Team 2013) program was used for computations and graphs were created by means of the ggplot2 program.

## Results

### Clinical observations

No animals and no samples were lost during the experiment. In general, post-surgical healing was uneventful.

### Qualitative and quantitative histology

**Fig. 1:**
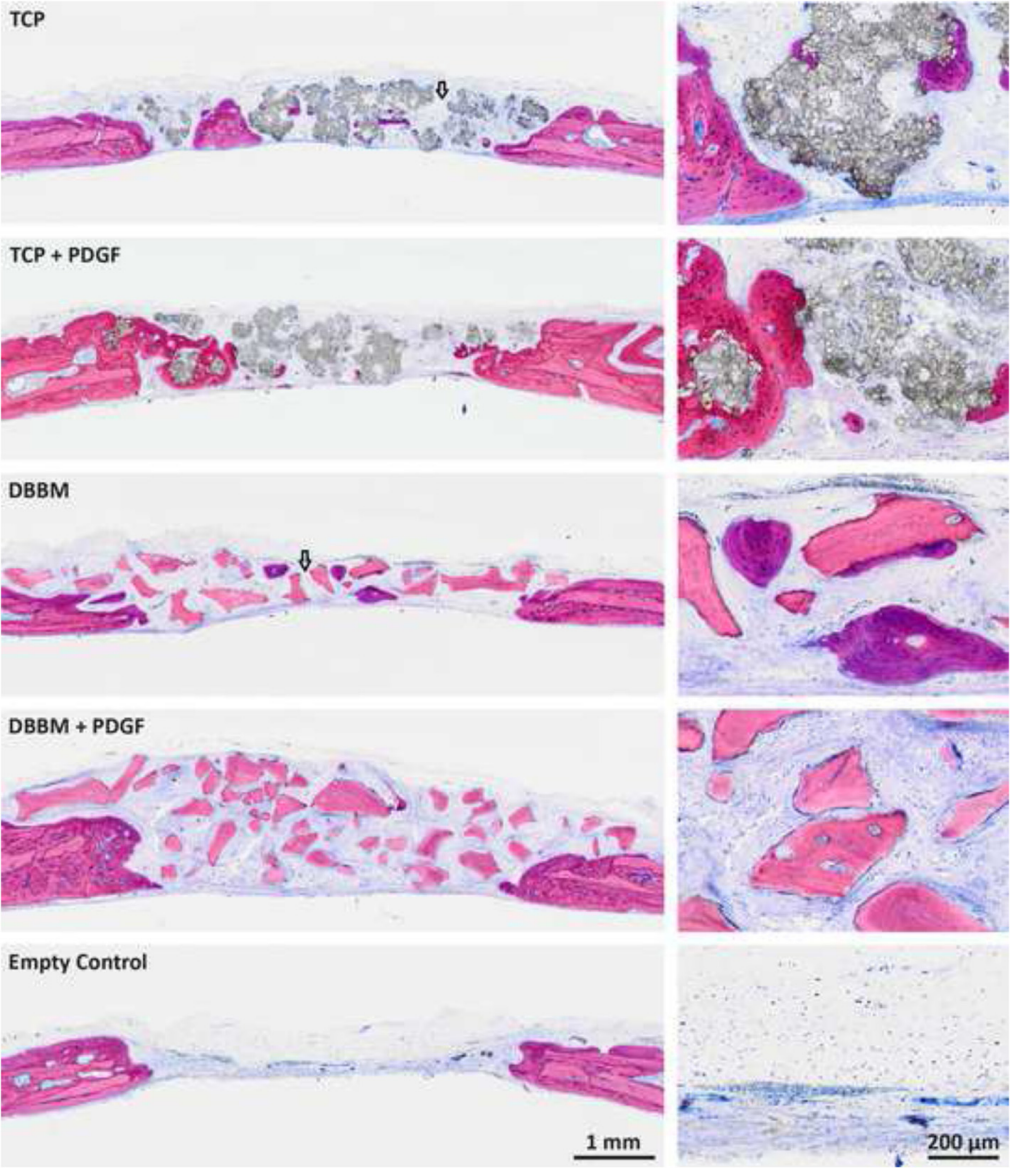
Histological images of treated and untreated rat calvarial defects at 45 days post-surgery: Representative images indicated partially healed defects, bridged by a conglomerate of biomaterial and new bone tissue for treated groups (TCP, TCP + PDGF, DBBM, DBBM + PDGF, Empty Control) (Levai-Laczko stain). New bone formation was mostly restricted to areas close to the original borders of defects in the control group. No substantial bone formation was observed in empty defects, only a thin layer of fibrous soft tissue separated the defect area from the underlying brain tissue. The empty control showed most of the defects occupied by connective tissue composed of large numbers of collagen fibers parallel to the wound defect. The defect sites that were originally treated with TCP and DBBM were partially bridged (arrow) by a conglomerate of biomaterial and new bone tissue. Immature osseous islands were also observed

As indicated in Fig. 1, no complete defect closure was observed in the histological specimens. No substantial bone formation was observed in empty defects; only a thin layer of fibrous soft tissue separated the defect area from the underlying brain tissue. New bone formation was mostly restricted to areas close to the original borders of defects in the control group. In empty controls, connective tissue composed of large numbers of collagen fibers parallel to the wound defect dominated the scene. The connective tissue in the central part of the defect was thinner than that in the original calvarium. Also noticeable in Fig. 1 were defect sites that were originally treated with TCP and DBBM; these were partially bridged by a conglomerate of biomaterial and new bone tissue. Immature osseous islands were occasionally observed. Remnants of TCP and DBBM were visible and in intimate contact with the bone tissue, especially at the defect margins. A similar situation occurred when TCP and DBBM were supplemented with PDGF-BB (Fig. 1). There were no obvious signs of cellular inflammatory infiltrate or foreign body reaction in most of the sections. A large portion of the graft particles remained unchanged and maintained the defect space. At the defect margins, new bone was laid onto the surface of TCP and DBBM, indicating that both biomaterials were biocompatible and osteoconductive.

**Fig. 2:**
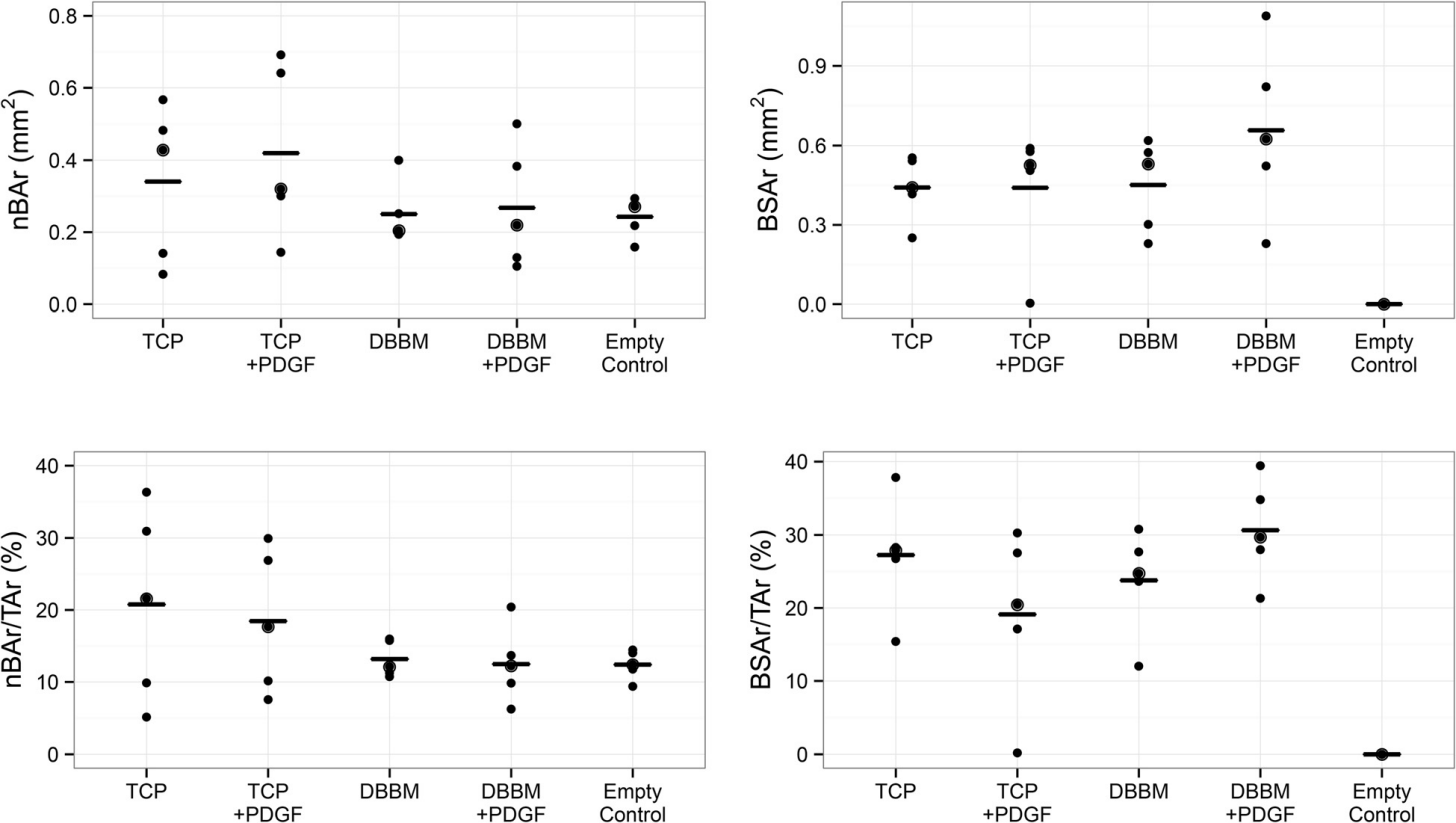
Histomorphometric analysis of the defect region. Area of newly formed bone in mm^2^ (nBAr); percentage of newly formed bone (nBAr/TAr); area of the remaining bone substitute material in mm^2^ (BSAr); percentage of bone substitute material (BSAr/TAr)

Histomorphometric analysis showed that nBAr at the defect site, and when normalized for the overall defect area (TAr), did not differ significantly among the five groups. Similarly, the residual amount of bone substitutes (BSAr) was not affected by supplementation with PDGF-BB (Fig. 2).

### Fluorescence staining

**Fig. 3:**
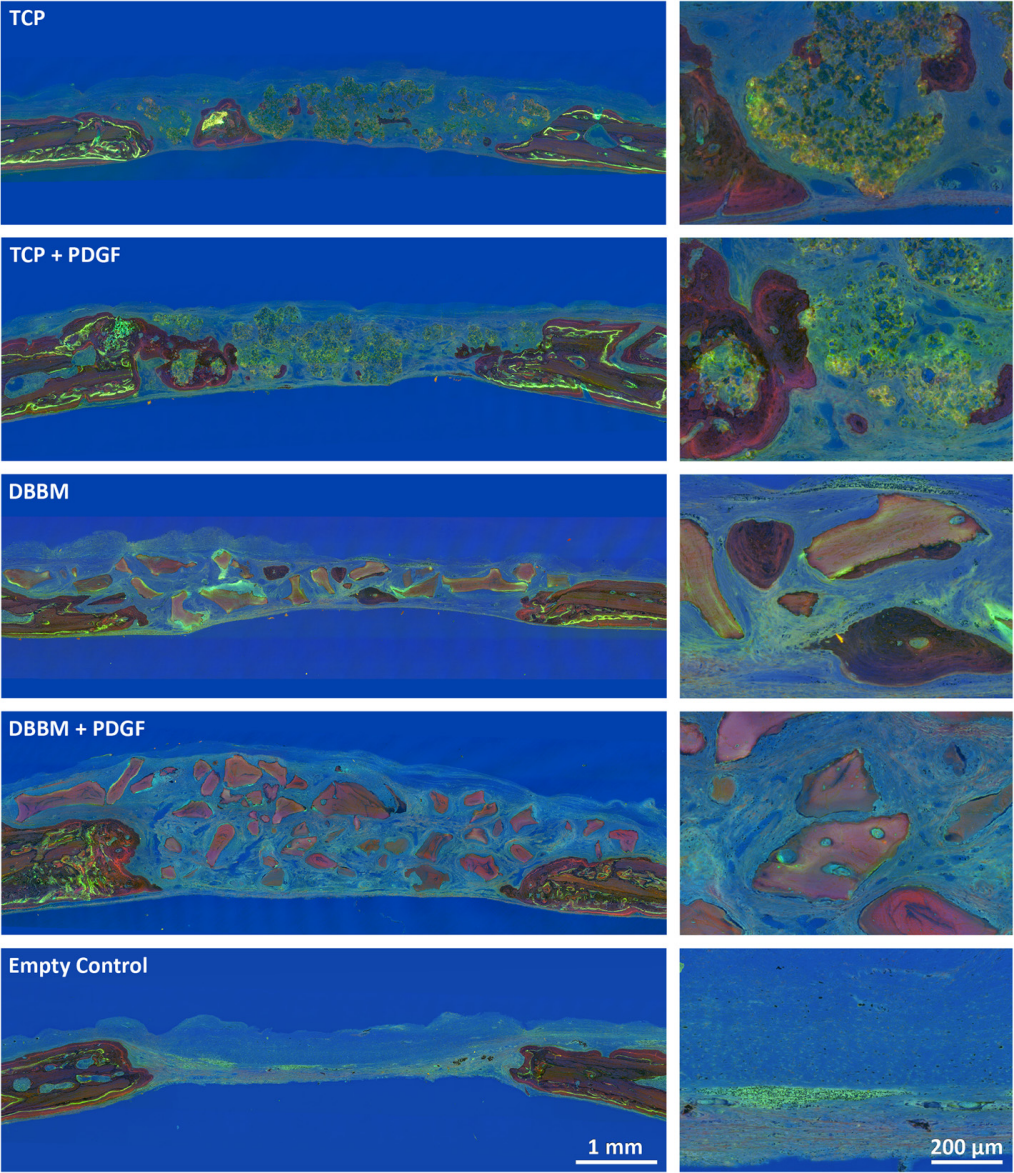
Fluorescence staining of treated and untreated rat calvaria defects: In agreement with the bright-field microscopic findings, bone formation, exhibiting green and red staining, was tenuous and occurred throughout the defects filled with TCP and DBBM, irrespective of the presence of PDGF-BB. No effects of PDGF-BB supplementation were observed. Empty control defects showed only weak staining that was also congruent with bright-field microscopy. These detailed analyses provided deeper insight into the osteoconductive properties of TCP and DBBM and the lack of visible changes after the addition of PDGF-BB

The distribution of green and red staining indicated bone formation between day 15 and day 30 post-surgery, irrespective of the biomaterial used (Fig. 3). Fluorochrome images at higher magnification support the histological findings; dark red staining in histological samples also emitted strong green and red fluorochrome signals. Bone formation (as indicated by green and red staining) occurred throughout the defects filled with TCP and DBBM, irrespective of the presence of PDGF-BB. Empty control showed only weak staining.

## Discussion

The key finding of the present study was that PDGF-BB had no impact on bone formation or the degradation of the two bone substitutes in rat calvarial defects at 45 days of defect healing. Consistent with these findings, PDGF-BB loaded onto TCP had no effect on new bone formation after 8 weeks in the rat calvarial defect model [17]. Furthermore, PDGF-BB combined with DBBM failed to improve bone formation after 3 and 5 months [18]. PDGF-BB in combination with allografts did not significantly increase bone formation in supracrestal defects in dog mandibles after 8 weeks [19]. The findings of the present study are also consistent with previous studies showing that PDGF-BB had limited impact on degradation of TCP particles over 4 weeks [20] and 8 weeks [17]. Although the majority of studies have found beneficial effects of PDGF-BB during bone regeneration [3, 8–11, 20, 21], it should be borne in mind that as yet, there are no clearly defined variables that predict the beneficial effects of PDGF-BB on bone regeneration.

The authors could speculate that the osteogenic capacity of rat calvarial defects is weak, probably because of the thin calvaria that is the origin of new bone. In support of this theory are the authors’ data showing no differences in the biomaterial residues between TCP and DBBM. These data were also unexpected because DBBM has a considerably slower degradation profile compared with TCP. Once again, the low turnover situation in rat calvarial defects may hold an explanation for this observation. Overall, bone healing for all groups was limited in this study, compared with other studies conducted with similar observation periods [16]. This low turnover situation was supported by the fluorescence staining results at 15 and 30 days post-surgery that were consistent with the histological observation of bone formation, again without visible changes arising from the addition of PDGF-BB. This low turnover situation is a possible explanation why the expected beneficial effects of PDGF-BB on bone regeneration failed in the present study. The rat calvarial defect model may perhaps not be ideal for studying the impact of PDGF-BB on bone formation in the presence of bone substitutes.

## Conclusions

In the pilot study, PDGF-BB loaded onto TCP or DBBM had no beneficial effect on new bone formation after 45 days in the rat calvarial defect model.

### Statement on ethics approval and consent

The present study complied with the principles of laboratory animal care and national laws on animal use, and the study was authorized by the Animal Research Ethics Committee of the Araçatuba Dental School, UNESP-Univ Estadual Paulista, São Paulo, Brazil (protocol # 02402-2012).

### Consent to publish

Not applicable.

### Availability of data and materials

Availability of data and materials section.

## Abbreviations

BSAr: Bone substitute material area.
nBAr: Newly formed bone (nBAr)
PDGF-BB: platelet-derived growth factor-BB
Tar: Tissue area
TCP: β-tricalcium phosphate
